# Concordance of immunohistochemistry for predictive and prognostic factors in breast cancer between biopsy and surgical excision: a single-centre experience and review of the literature

**DOI:** 10.1007/s10549-023-06872-9

**Published:** 2023-02-21

**Authors:** Chiara Rossi, Sara Fraticelli, Marianna Fanizza, Alberta Ferrari, Elisa Ferraris, Alessia Messina, Angelica Della Valle, Chiara Annunziata Pasqualina Anghelone, Angioletta Lasagna, Gianpiero Rizzo, Lorenzo Perrone, Maria Grazia Sommaruga, Giulia Meloni, Silvia Dallavalle, Elisabetta Bonzano, Marco Paulli, Giuseppe Di Giulio, Adele Sgarella, Marco Lucioni

**Affiliations:** 1 Department of Molecular Medicine, Unit of Anatomic Pathology, University of Pavia, IRCCS San Matteo Hospital Foundation, Pavia, Italy; 2 Unit of Breast Radiology, IRCCS San Matteo Hospital Foundation, Pavia, Italy; 3 Department of Surgical Sciences, General Surgery 3—Breast Surgery, IRCCS San Matteo Hospital Foundation, Pavia, Italy; 4 Unit of Medical Oncology, IRCCS San Matteo Hospital Foundation, Pavia, Italy; 5 School in Experimental Medicine, Unit of Radiational Oncology, University of Pavia, IRCCS San Matteo Hospital Foundation, Pavia, Italy

**Keywords:** Breast cancer, Preoperative diagnosis, Immunohistochemistry, Biomarkers, Er-low-positive, her2-low

## Abstract

**Purpose:**

Accurate evaluation of breast cancer on bioptic samples is of fundamental importance to guide therapeutic decisions, especially in the neoadjuvant or metastatic setting. We aimed to assess concordance for oestrogen receptor (ER), progesterone receptor (PR), c-erbB2/HER2 and Ki-67. We also reviewed the current literature to evaluate our results in the context of the data available at present.

**Methods:**

We included patients who underwent both biopsy and surgical resection for breast cancer at San Matteo Hospital, Pavia, Italy, between January 2014 and December 2020. ER, PR, c-erbB2, and Ki-67 immunohistochemistry concordance between biopsy and surgical specimen was evaluated. ER was further analysed to include the recently defined ER-low-positive in our analysis.

**Results:**

We evaluated 923 patients. Concordance between biopsy and surgical specimen for ER, ER-low-positive, PR, c-erbB2 and Ki-67 was, respectively, 97.83, 47.8, 94.26, 68 and 86.13%. Cohen’s κ for interobserver agreement was very good for ER and good for PR, c-erbB2 and Ki-67. Concordance was especially low (37%) in the c-erbB2 1 + category.

**Conclusion:**

Oestrogen and progesterone receptor status can be safely assessed on preoperative samples. The results of this study advise caution in interpreting biopsy results regarding ER-low-positive, c-erbB2/HER and Ki-67 results due to a still suboptimal concordance. The low concordance for c-erbB2 1 + cases underlines the importance of further training in this area, in the light of the future therapeutic perspectives.

## Introduction

Assessment of breast cancer biomarkers has become a staple of routine histopathology for every colleague working in this field. Assessment and quantification of oestrogen receptor (ER), progesterone receptor (PR), and c-erbB2/HER2 are used daily by the clinicians making fundamental therapeutic choices for the patients. On the other hand, Ki-67 has struggled to join this established trifecta in the routine management and risk stratification of breast cancer patients [1–4] and its prognostic and predictive value is restricted to very specific settings in breast cancer; recently, the results from the monarchE study has suggested a prognostic role for Ki-67 ≥ 20% in patients with early breast cancer treated with cyclin-dependent kinase 4 and 6 (CDK4/6) inhibitors in combination with endocrine therapy [5].

Recently, changes in the stratification of ER positivity have been put forward by ASCO/CAP through an update of the recommendations for ER and PR determination, with the formal introduction of the ER-low-positive (ER-LP, defined as 1–10% of nuclear positivity in the tumour) [6]; this category has been known for some time to share more similarities with basal-type triple-negative breast cancer than with the luminal group [7–10], in morphological aspects, molecular signature and clinical behaviour.

In the same way, the results of the DESTINY-Breast03 trial [11, 12], and the identification of the HER2-low category [13, 14] as a new group of patients who may significantly benefit from anti-HER2 target therapy administration, have underlined once again the importance of the c-erbB2 status and its assessment with both immunohistochemistry and molecular techniques.

Core needle biopsy (CNB) is the most common method for diagnosis of breast cancer, and it has been demonstrated to be a reliable indicator of the surgical specimen results [15, 16]. Assessment of biomarkers on the preoperative specimen is required to administer neoadjuvant therapy to the selected patients that benefit from it and, in case of a complete pathological response, it represents the only sample of that tumour available; also for metastatic patients, who are not eligible for surgical resection, the bioptical sample is the only one that can be used to take life-changing clinical decisions.

It is clear, then, the importance of a reliable assessment of these biomarkers on biopsy. Given the steadily increasing request for precise molecular characterization of breast cancer to ensure correct patient management, we aimed to retrospectively evaluate our patient cohort for reproducibility of the biopsy results for ER, PR, c-erbB2, and Ki-67, focusing also on the recently defined ER-LP and HER2-low groups. We also review the recent literature on the topic to validate our results in the context of the international available results.

## Methods

### Patients and clinicopathological characteristics

We reviewed the records of 1654 breast cancer patients who underwent both biopsy and surgical excision at the Department of Breast Surgery, San Matteo Hospital, between January 2014 and December 2020. We excluded patients for which ER, PR, c-erBb2 or Ki-67 values were missing, patients with in situ or microinvasive-only breast cancer, patients with multifocal tumours, metastatic disease and patients who underwent neoadjuvant chemotherapy. Our final cohort comprised 923 patients and their clinical characteristics are summarized in Table 1. The study was conducted according to the guidelines of the Declaration of Helsinki.

**Table 1:** Clinicopathological characteristics of the cohort

Characteristic	*n* (%)
Sex	
Female	918 (99.4)
Male	5 (0.6)
Median age	55
Histology	
No special type	707 (77)
Lobular	126 (14)
Other special histotypes	90 (9)
Grade	
G1	76 (8)
G2	537 (58)
G3	310 (34)

### Pathology evaluation

Samples were fixed in 10% neutral buffered formalin and embedded in paraffin before histopathological evaluation. 4-to-5 µm-thick sections were cut and stained with hematoxylin and eosin (HE), and unstained sections were used for immunohistochemistry with antibodies anti-ER (clone EP1, Dako Omnis), anti-PR (clone PgR 1294, Dako Omnis), c-erbB2 (clone A0485, Dako Omnis) and Ki-67 (clone MIB-1, Dako Omnis). All immunoreactions were carried out on a Dako Omnis platform (Dako, Glostrup, Denmark). All cases were seen by at least one pathologist (M.L. and/or C.R.) expert in breast pathology, who also revised all the discrepant cases prior to the final diagnosis.

ER and PR were defined positive when ≥ 1% of the tumour cell nuclei showed immunostaining, according to the 2010 ASCO/CAP guidelines [17]. ER was further stratified into LP and positive using the 10% cut-off, according to the 2020 ASCO/CAP guidelines update [6]. Ki-67 was scored ‘high’ when ≥ 20% of the tumour nuclei were positive, taking into account the cut-off clinically used to define the Luminal B class according to the 2013 St Gallen International Breast Cancer Conference experts Panel opinion [18]. Cells positive for Ki-67 were scored over 100 cells in both ‘cold’ and ‘hot’ tumour areas, and the final value represented an average between those of the different areas. This method is similar to the one recommended by the International Ki-67 in Breast Cancer Working Group (IKWG) in their 2021 updated recommendations [19], with the only difference that the recommended online scoring app was not used. c-erbB2 was scored according to the ASCO/CAP 2013 guidelines and 2018 Focused Update [20, 21] as 0, 1 +, 2 + or 3 + depending on intensity and completeness of the membrane staining. FISH was performed in all the equivocal cases, but the results are not reported in this paper since we focus on the immunohistochemical evaluation alone.

Cohen’s kappa (κ) was used to measure the interobserver agreement between biopsy and surgical specimen; weighted κ was used when concordance between more than one result was evaluated to account for close matches. *κ* values < 0.20 were interpreted as poor agreement, 0.21–0.40 fair agreement, 0.41–0.60 moderate agreement, 0.61–0.80 good agreement, 0.81–0.99 very good agreement and 1 perfect agreement. P-values were calculated with Fisher’s exact test and chi-squared test, when appropriated, on GraphPad Prism 5, and *p*-values < 0.05 were deemed significant.

## Results

### ER

ER was positive in 829/923 (89%) biopsies and in 831/923 (90%) surgical resection specimens, with a concordance of 97.83% (*n* = 903/923), a Cohen’s *κ* of 0.880 (very good agreement) and a *p*-value < 0.0001. The concordant and discrepant results are reported in Table 2, with 9 positive results (9/923, 1%) later reported as negative on the surgical specimen and 11 negative results (11/923, 1.2%) that were upgraded to positive on the final pathology report. These changes would have had a clinical impact with either withholding or administration of endocrine therapy in these patients, if the decision was based only on the biopsy results.

**Table 2:** Concordance between biopsy and surgical specimen for oestrogen receptor, progesterone receptor and Ki-67. ER, oestrogen receptor, PR, progesterone receptor

Biopsy	Surgical specimen		Total
ER	+	−	
+	820	9	829
–	11	83	94
PR			
+	742	17	759
−	36	128	164
Ki-67			
+	235	21	256
−	107	560	667

**Fig. 1:**
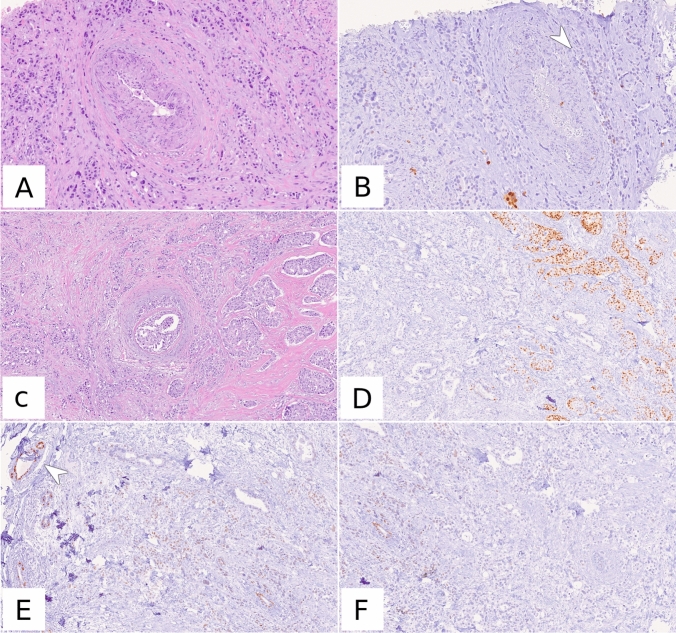
Example of ER-LP discordance between biopsy and surgical specimen. On biopsy, (**A**, HE, 10x, **B**, ER immunohistochemistry, 10x) the tumour showed only faint, very focal (arrowhead) positivity for ER, that was quantified at 1%. The surgical specimen (**C**, HE, 5x, **D**–**F**, ER immunohistochemistry, 5x) revealed a dishomogeneous and faint ER positivity. Note the positive internal control (arrowhead) in the top-left corner of panel (**E**)

When the positive results were further stratified into ER-LP and ER-positive (Table 3) the overall agreement was still very good (97.18%, weighted *κ* = 0.924, *p* value < 0.0001), but the concordance for the ER-LP category itself was low (*n* = 11/23, 47.8%). Of the 12 discordant ER-LP results on biopsy, 8/12 (66.7%) were ER-negative on the surgical specimen and 4/12 (33.3%) were ER-positive. Of these four cases, three cases were only slightly above the cut-off for ER-LP (15%), whilst one showed positivity in 60% of the cells (Fig. 1).

**Table 3:** Concordance between biopsy and surgical specimen for oestrogen receptor-negative, oestrogen receptor-LP and oestrogen receptor-positive cases. ER, oestrogen receptor

Biopsy	Surgical specimen			Total
	ER-negative	ER-LP	ER-positive	
ER-negative	83	10	1	94
ER-LP	8	11	4	23
ER-positive	1	2	803	806

### PR

PR was positive in 759/923 (82%) biopsies and in 778/923 (84%) surgical resection specimens. Concordance was 94.26% (*n* = 870/923), Cohen’s κ was 0.794 (good agreement) and *p* value was < 0.0001. Most (*n* = 36/53, 67.9%) of the discordant results were biopsies reported as PR negative that were later reported as PR positive on the surgical specimen. The complete results are summarized in Table 2.

### c-erbB2/HER2

c-erbB2 was found to be concordant in 68% of cases (*n* = 631/923), and Table 4 details the results for each category. Cohen’s weighted *κ* was 0.675 (good agreement) and *p*-value was < 0.0001. Breaking down the results for each category, 1 + was the least concordant group (37% vs 83%, 79% and 97% for 0, 2 + and 3+ , respectively), with 72% (*n* = 136/188) of the discordant results being diagnosed as 0 on the surgical specimen.

**Table 4:** Concordance between biopsy and surgical specimen for c-erbB2 status

Biopsy	Surgical Specimen				Total
	0	1 +	2 +	3 +	
0	321	54	14	0	389
1 +	136	112	50	2	300
2 +	2	29	130	3	164
3 +	0	2	0	68	70

According to current clinical practice, only four (4/923, 0.4%) patients had changes in the diagnosis that could significantly impact their treatment choice (two 1 + biopsies later upgraded to 3 and two 3 + biopsies downgraded to 1 +). They were all, except for one locally advanced cancer, early breast cancers that would have not been candidates for neoadjuvant treatment. However, on the account of the results of the biopsy alone, they would have been denied a potentially life-saving treatment or subjected to the toxicities of an ineffective one.

### ki-67

Ki-67 was high in 256/923 (28%) biopsies and in 357/923 (38%) surgical resection specimens. Concordance was 86.13% (*n* = 759/923), Cohen’s *κ* was 0.686 (good agreement), and *p* value was < 0.0001. Concordance and discordance are reported in Table 2; most of the discordant results (*n* = 107/128, 84%) were biopsies in which Ki-67 was reported as low that were later upgraded on the excision specimen.

## Discussion

### ER

In the published literature ER concordance between biopsy and surgical excision averages at 93.3% (range 78.7–99.1%, Table 5). Our concordance was 97.83%, slightly higher than the average value but in keeping with the known results. This variance can also be attributed to the different cut-offs and scoring methods used in the single laboratories, reported in the table that impair their universal reproducibility.

**Table 5:** Review of the literature regarding oestrogen receptor concordance

	No of samples	Concordance (%)	IHC clone
Badoual, 2005 [25]	103	90.3	1D5, Immunotech
Burge, 2006 [26]	87	95	SF11, Biocare Medical
Hodi, 2007* [27]	338	98.8	1D5, Dako
Wood, 2007 [28]	100	95.8	ID11, Dako
Arnedos, 2009 [29]	336	98.2	SP1, Ventana
Tamaki, 2010 [30]	353	94.9	SF11, Ventana
Uy, 2010§ [31]	160	81.9	1D5, Dako
Lorgis, 2011° [32]	175	84	6F11, Novocastra
Ough, 2011 [33]	209	88	Not reported
Li, 2012# [34]	2450	92.8	
Seferina, 2012° [35]	526	89.5	1D5, Dako
Dekker, 2013 [36]	122	99.1	1D5, Dako
Greer, 2013 [37]	205	89	6F11, Leica 1D5, Dako
Munch-Petersen, 2013 [38]	89	98	SP1, Dako
Motamedolshariati, 2014 [39]	30	96.7	Not reported
Chen, 2017 [40]	996	78.8	SP1, Ventana
Ensani, 2017 [41]	100	90	1D5, Biogenex
Kombak, 2017 [42]	284	93.3	6F11, Leica
Meattini, 2017° [43]	101	94.1	SF11, Ventana
You, 2017§ [44]	1219	97.1	SF11, Novocastra
Berghuis, 2019 [45]	684	95.5	Multiple
Jeong, 2020§ [46]	623	96.5	SP1, Ventana
Shanmugalingam, 2022 [47]	484	96.7	SP1, Ventana
Slostad, 2022 [48]	961	90.8	LDT

*McCarty’s h scoring system, #meta-analysis, ° positivity threshold: 10%, § Allred score, IHC, immunohistochemistry, LDT, laboratory-developed test.

Several factors are recognized to influence concordance between biopsy and surgical specimen, with the pre-analytical phase being the most important to standardize intra-laboratory results, trying to avoid under-fixation [17, 22]. The choice of the antibody clone and the immunostaining platform has also been demonstrated to be relevant in the over- or underestimation of the ER percentage [23], especially when comparing the widely used Dako and Ventana clones.

The high percentage (66.7% of ER-LP cases) of ER-LP that tested ER-negative on the surgical specimen suggests that ER-LP results more frequently represent an “overcalling” of a non-luminal carcinoma, in line with the current understanding of ER-LP tumour biology, that relates them more closely to this group. Recent published data suggest also that artefactual reduced intensity of staining of ER in normal breast tissue adjacent to the neoplasia may concur to report these cases as ER-LP [24], therefore underlying the importance of the presence of internal and/or on-slide controls in the assessment of these borderline cases.

In summary, our results suggest that caution should be taken when calling ER-LP on biopsy and for subsequent decision-making, and further studies are needed to define if this category can be safely defined on a biopsy specimen.

### PR

Our final concordance for PR was 94.26%. From a review of the literature, the average reported concordance is 87.3% (range 73.5–95%, Table 6), and our results fall on the upper side of this range.

**Table 6:** Review of the literature regarding PR concordance

	No of samples	Concordance (%)	IHC clone
Badoual, 2005 [25]	103	89.3	PR10A9, Immunotech
Burge, 2006 [26]	87	89	PgR 636, Dako
Wood, 2007 [28]	100	90.3	PgR 636, Dako
Arnedos, 2009 [29]	336	85	1E2, Ventana
Tamaki, 2010 [30]	353	89.5	6, Ventana
Uy, 2010§ [31]	160	85.6	PgR 636, Dako
Lorgis, 2011° [32]	175	78.3	PgR 636, Dako
Ough, 2011 [33]	209	78	Not reported
Li, 2012# [34]	2448	84.8	
Seferina, 2012° [35]	526	83.6	PgR 636, Dako
Greer, 2013 [37]	205	89	PgR 1294, Dako PgR 636, Dako
Motamedolshariati, 2014 [39]	30	90	Not reported
Chen, 2017 [40]	985	73.5	1E2, Ventana
Ensani, 2017 [41]	100	81	PR88, Biogenex
Kombak, 2017 [42]	284	89.4	16, Leica
Meattini, 2017° [43]	101	88.1	16, Ventana
You, 2017§ [44]	1219	95	16, Novocastra
Berghuis, 2019 [45]	890	84.8	Multiple
Jeong, 2020§ [46]	623	93	1E2, Ventana
Shanmugalingam, 2022 [47]	484	93.2	IE2, Ventana
Slostad, 2022 [48]	961	87.2	LDT

# meta-analysis, ° positivity threshold: 10%, § Allred score, *IHC* immunohistochemistry, *LDT* laboratory-developed test

The lower concordance of PR assessment on biopsy and surgical specimen reflects its naturally occurring dishomogeneity in breast normal tissue and tumours, owing to his nature as a down-stream ER effector and therefore requiring an intact ER pathway to be strongly expressed. Our results with a higher proportion of upgrades rather than downgrades on the surgical specimen (67.9% vs 32.1%) suggest that indeed tumour heterogeneity, with a negative spot sampled on biopsy, may be a significant issue in PR assessment.

Given this heterogeneity, PR concordance is especially sensitive to sampling artefacts, especially undersampling of the target lesion. From the current literature, four represents the minimum number of biopsy cores that should be retrieved to ensure a correct preoperative evaluation [49].

### c-erbB2/HER2

In the published literature, concordance for c-erbB2 when evaluated with immunohistochemistry alone averages at 85.4% (range 56–98.8%, Table 7); our series demonstrates a concordance in 68% of cases, lower than the reported values, but still in the published range.

**Table 7:** Review of the literature regarding c-erbB2/HER2 concordance

	No of samples	Concordance (%)	IHC
Burge, 2006 [26]	81	96	Dako
Wood, 2007 [28]	100	86.6	Dako
Arnedos, 2009 [29]	331	98.8	Ventana
Lebeau, 2010 [51]	500	90,4	Dako
Tamaki, 2010 [30]	353	89.3	Ventana
Lorgis, 2011* [32]	175	98.3	Ventana
Ough, 2011 [33]	209	56	Not reported
Lee, 2012* [52]	300	98	Dako
Seferina, 2012° [35]	526	80.6	Dako
Dekker, 2013 [36]	122	96.4	Dako
Munch-Petersen, 2013 [38]	89	84.0	Ventana
Motamedolshariati, 2014 [39]	30	93.3	Dako
Chen, 2017 [40]	941	62.6	Ventana
Ensani, 2017 [41]	100	97.3	Not reported
Kombak, 2017 [42]	243	90.1	Leica
You, 2017 [44]	1219	84.6	Ventana
Slostad, 2022 [48]	961	73.4%	LDT

*IHC + FISH, °IHC + SISH (Ventana), *IHC* immunohistochemistry, *LDT* laboratory-developed test

Breaking down the results, the 1 + category was the one with the lowest concordance, with only 37% of results getting confirmation on the surgical specimens. Whilst we had previously reported that this discordance for the 1 + category was not likely to have a significant therapeutic impact for the patients [50], the recent introduction of the HER2-low category as a subset of patients that could benefit from the administration of targeted anti-HER2 therapy will radically change that in the upcoming years.

The 2013 ASCO/CAP guidelines define 1 + as incomplete membrane staining that is faint or barely perceptible and within > 10% of tumour cells and readily appreciated in a contiguous population using a low-power objective, whereas 0 is defined as absence of staining or incomplete membrane staining that is faint/barely perceptible and within ≤ 10% of tumour cells [20]. This diagnosis must be made on immunohistochemistry alone, with no help coming from ancillary molecular studies.

However, in everyday practice, this distinction is not an easy one to make especially on biopsy, where crushing or technical artefacts and tumour heterogeneity make it a particularly challenging task. In the light of the growing importance of distinguishing between 0 and 1 +, we stress the importance of a dedicated, up-to-date breast pathologist examining these specimens, especially in those difficult cases in which the diagnosis is not immediately clear.

### Ki-67

Ki-67 represents an unfulfilled promise in the field of breast cancer; despite being widely used as a marker of proliferation, it has failed time and time again to reach prognostic and predictive significance.

This is at least in part ascribable to the still unclear nature of this biomarker that, despite the best efforts that has been put into it, still does not have a fixed biological cut-off; guidelines for what qualifies as ‘high’ and ‘low’ Ki-67, and whether these definitions truly represent different biological entities, are still unclear.

Recent recommendations from the IKWG [19, 53] report that sufficient levels of evidence of the prognostic value of Ki-67 exists only in the setting of ER-positive early-stage breast cancer, where levels ≥ 5% and ≥ 30%, respectively, may favour withholding or administration of chemotherapy. Historically, the 2013 St Gallen International Breast Cancer Conference suggested a 20% cut-off for the definition of ‘high’ Ki-67 in the definition of the surrogate intrinsic subtypes of breast cancer [18], but still advised that different cut-offs could be adopted by single laboratories. As reported in literature [40, 43, 44, 46, 54, 55], most laboratories use either a 14% or a 20% cut-off.

Our concordance was in line with the values reported in literature (Table 8), especially with those reported by You et al. [44], whose cut-off and population are similar to those of this work. Similar values are reported also with a 14% cut-off by Kim et al., [55], Ahn et al., [54] and Meattini et al. [43], whilst lower concordance levels are reported by studies in which a Ventana antibody is used for immunohistochemistry [40, 46] irrespectively of the cut-off used.

**Table 8:** Review of the literature regarding Ki-67 concordance

	No of samples	Concordance (%)	IHC clone
Ough, 2011 [33]	209	59	Not reported
Greer, 2013 [37]	205	73	MIB-1, Dako
Kim, 2016* [55]	310	85.8	MIB-1, Dako
Ahn, 2017* [54]	89	82	MIB-1, Dako
Chen, 2017* [40]	696	70.3	30–9, Ventana
Kombak, 2017* [42]	236	80.9	K2, Leica
Meattini, 2017* [43]	101	88.1	Mib-1, Immunotech
You, 2017§ [44]	1219	87	MIB-1, Dako
Jeong, 2020§ [46]	623	78.7	MIB-1, Ventana
Shanmugalingam, 2022# [47]	484	70.5	Not reported

*Cut-off high expression: 14%, § cut-off high expression: 20%, #, stratified into three categories (low, intermediate, high), IHC, immunohistochemistry

To our knowledge, this work represents one of the largest single-centre series present in literature and the first one where issues within the ER-LP and HER2-low categories were specifically addressed. Moreover, all the immunohistochemistry reactions were carried out with the same antibodies on the same platform, and a breast pathologist (M.L. and/or C.R.) was involved in the diagnosis of all cases, ensuring a high degree of homogeneity in the test results. Possible limitations include the retrospective nature of the study and the lack of data regarding the HER2 2 + amplification status; however, this study aimed to evaluate only the immunohistochemical concordance for HER2, with particular attention to the HER2-low categories.

We confirmed a very good agreement for ER assessment on biopsy, and a still satisfactory, albeit lower, concordance for PR that falls just short of the very good agreement cut-off. Ki-67 evaluation instead was confirmed to be slightly less reliable than ER and PR, with a significantly lower concordance, and could warrant evaluation also on the surgical specimen, if available, in the light of potential treatment options [5].

ER-LP analysis revealed that, even if the global ER concordance is still satisfactory, the concordance for the specific category is still low and must be further investigated to define the boundaries within which it can be safely assessed on a biopsy sample.

The low concordance for the 1 + c-erbB2 immunohistochemistry is particularly relevant in the context of the new therapeutic advances involving it and highlights the technical difficulties in consistently implementing the current diagnostic criteria available for the diagnosis.

In the light of the quick and exciting evolution of the therapeutic landscape of breast cancer, it is important that caution is taken in evaluating biopsy samples, especially for those predictive factors that may significantly impact the therapeutic options of the patient. Although only a very small number of patients in our series would have received an inappropriate treatment based on biopsy results alone, our data underline the relevance of surgical sample retesting, at least in selected cases, including large tumours and cases with discrepant histological characteristics. Specific training needs should be addressed by the national and international pathology societies, especially in those still grey areas of ER-LP and HER2-low categories, where the difference between a negative and a positive result may withhold a target therapy, or cause patients to undergo unnecessary, and often quite burdensome, aggressive therapy.

## Data Availability

The original contributions presented in this study are included in the article. Further inquiries can be directed to the corresponding author.
